# Feedback matters: EEG correlates of empathy involved in the naturalistic communication of emotions

**DOI:** 10.1016/j.heliyon.2024.e38574

**Published:** 2024-09-27

**Authors:** Ruei-Jyun Hung, Intan Low, Hung-Chun Yeh, Po-Yu Wang, Yong-Sheng Chen, Li-Fen Chen

**Affiliations:** a Institute of Brain Science, National Yang Ming Chiao Tung University, Taipei, Taiwan; b Department of Computer Science, National Yang Ming Chiao Tung University, Hsinchu, Taiwan; c Institute of Biomedical Informatics, National Yang Ming Chiao Tung University, Taipei, Taiwan

**Keywords:** Feedback, Empathy, Alpha activity, Electroencephalography

## Abstract

Empathy involves the processing of complex information related to dynamic interactions between the empathizer and target. One neural signature of empathy is the suppression of electroencephalographic mu rhythm (8–13 Hz) over the sensorimotor region. It is important to consider that few researchers have studied the effects of empathizer feedback on empathy and its underlying neural mechanism, and most previous research has lacked ecological validity due to standardized emotional stimuli and constraints on the experiment environment. Our objective in this study was to investigate the means by which empathizer feedback influences one's own empathy in naturalistic social situations. Our results revealed that empathizer feedback decreases empathic accuracy but does not affect the emotional contagion nor the emotional intensity of the empathizer. We also found that the ability to accurately infer sadness is hindered by empathizer feedback. Empathizers presented lower alpha activity in the sensorimotor cortical areas only while receiving sad narratives and not providing feedback. This study contributes to the emerging research on the influence of empathizer feedback in naturalistic social settings.

## Highlights

• Empathizer feedback does not affect emotional contagion.
• The ability to accurately infer sadness is hindered by empathizer feedback.
• Reduced alpha activity in the sensorimotor cortex to sadness in empathizers without feedback.

## Graphical abstract

**Figure:**
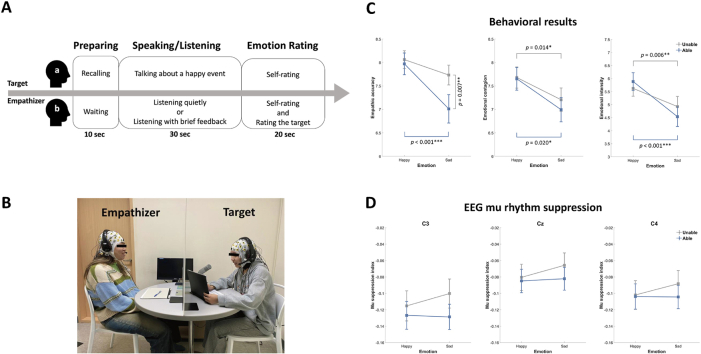

## 1 Introduction

Conversation is an important form of social communication in human beings. Through narratives, facial expressions, or gestures, individuals can comprehend others' intentions and emotional states, leading to further prosocial actions like providing assistance or strengthening social bonds [1,2]. Despite the significance of passively receiving information [[3], [4], [5], [6]], recent studies have indicated that the empathy process is profoundly affected by the interaction between the empathizer (who observes emotional expression) and the target (who expresses emotional feelings, such as distress) [7]. “Adaptive empathy” was defined by Shamay-Tsoory and Hertz [7] to refer to an empathizer's ability to adapt their response through observing a target’s distress level. Appropriate response from an empathizer is crucial to the smooth flow of social interactions [8]. Listener responses were found to reduce the negative emotions [9] and the activity of the reward system of the target [10]. However, little is known about the effects of listener responses on the formation of empathy in observers.

The generation of empathy in observers is influenced by emotional triggers, such as emotional valence, and observer factors, such as relationships and age, in interactions [11]. For example, individuals tend to show higher levels of empathy towards pleasant situations rather than unpleasant ones [12], towards their friends compared to strangers [13], and younger women tend to exhibit higher levels of empathy compared to older women [12]. Although several observer factors have been investigated, whether and how the observers’ behavior in interactions influences their empathy remains unclear.

Recently, second-person neuroscience has uncovered neural mechanisms underlying social interaction using dual-brain approaches [14,15]. An increasing number of studies have investigated empathy [9,[16], [17], [18]], communication [[19], [20], [21], [22], [23], [24]], and interpersonal relationships [[25], [26], [27]]. Toppi et al. [16] found that the synchronization of event-related potentials was related to the positive empathy of the receiver and observer using a third-party punishment task. Anders et al. [17] reported that mutual activation between the empathizer's and the target's brains was related to successful emotion recognition, revealing the neural representation of interpersonal understanding. Wang et al. [9] found that active listening improved interpersonal emotional regulation and increased interbrain synchronization in the brain regions related to emotion and language, implicating that active listening of the empathizer may play an important role in interpersonal emotion regulation. Although a growing number of studies have put effort into interpersonal interaction and empathy, whether and how empathizer response influences empathic responses remains unclear.

Listener responses (also known as backchannels) can be designated as generic or specific [28]. Generic responses are those that do not contain specific information related to the narrative, including verbal (such as “hmmm”) and nonverbal (such as nodding) displays of active engagement [8,28]. Specific responses are those that connect to the content of the narrative, such as facial expressions of sadness or fear. Bavelas et al. [28] found that distracted listeners (i.e., performing a word-counting task while receiving the narrative) made fewer generic and specific responses, which appeared to hinder the ability of the speaker to present a cogent narrative. Research has shown that active listening with corresponding cues indicating genuine responses and unconditional positive regard could have a profound effect on regulating interpersonal communication and the corresponding emotional responses [29,30]. Despite solid evidence that listener responses influence the target, little is known about whether or how empathizer feedback influences one's own empathy.

Although previous research on empathy largely focused on vicarious negative emotions, an increasing number of studies have indicated the importance of sharing positive emotions [31]. Empathy associated with negative emotions (also known as “negative empathy” or “empathic concern”) has played a key role in the survival of human groups (e.g., through the provision of care for offspring or the infirm) [32,33]. Empathy associated with positive emotions (also known as “positive empathy” or “empathic happiness”) is fundamental to interpersonal relationships and group cohesiveness [31]. One study in the field of social neuroscience reported that people tend to exhibit stronger brain responses to distress than to joy [34]. Recent second-person neuroscience studies indicated that sharing happiness facilitates emotional contagion, interpersonal closeness, coordination, and interbrain synchrony [[35], [36], [37]]. Specifically, Xie et al. [37] found that sharing happy stories increased interpersonal closeness and interbrain synchrony more than sharing sad stories, suggesting that sharing positive emotions is more helpful in enhancing speaker-listener interaction. Several studies have also reported that the neural bases of positive and negative empathy are fundamentally different [[38], [39], [40]]. Empathy for negative emotions is associated with the anterior insula and dorsal anterior cingulate cortex, while empathy for positive emotions is linked to the ventromedial prefrontal cortex and ventral striatum [38,41]. Taken together, it appears that gaining a thorough understanding of the relationship between empathizer feedback and empathy will require an analysis of the effect in the context of negative as well as positive empathy.

Empathy for negatively and positively valenced emotions has been investigated, yet there are mixed findings. Kelly et al. [42] revealed that the contagion of anger is automatic and not affected by increased cognitive load, while the contagion of happiness is reduced by increased cognitive load. Morelli and Lieberman [4] found no difference in self-reported empathy for happiness and sadness. Conversely, Mackes et al. [43] demonstrated better recognition for happy videos compared to sad ones. The inconsistent results may stem from the limitations of focusing on specific aspects of empathy. A recent study [44] suggested that both emotional valence and cognitive/emotional components of empathy should be considered when assessing empathy.

Empathy has been investigated with various methods due to its diverse definitions [45,46]. It is a multifaceted social process comprising emotional and cognitive components [47,48]. Emotional empathy (also known as “experience sharing” and “affective empathy”) is a bottom-up process involving a resonance with the emotional states of others (e.g., I feel your pain) [45,47]. Cognitive empathy (also known as “mentalizing” and “perspective-taking”) is a top-down process of inference pertaining to the intentions, beliefs, and/or thoughts of others (e.g., I know you are in pain) [45,47]. Previous studies employed the mere self-report of an empathizer and the congruence between an empathizer and the target to investigate empathy. The former includes Emotional Intensity (EI), which measures an empathizer's emotional state [40,49]. For example, if a participant self-reported the absence of sadness after viewing a sad video clip, they would be considered to show low emotional empathy. The latter includes Emotional Contagion (EC) and Empathic Accuracy (EA). Emotional contagion, which measures the congruence between the empathizer's and the target's emotional states, was considered an indicator of emotional empathy [45]. Empathic accuracy (EA) measures the accuracy with which the empathizer judges the emotional state of the target and is considered an index of cognitive empathy [3,50]. Both EI and EC reflect emotional resonance and are therefore considered indices of emotional empathy [5,51]. Given the intricate relationship between emotional valence and empathy components, this study employs three empathy indices (EA, EC, and EI) to explore the impact of empathizer feedback on a multidimensional conceptualization of empathy for positively and negatively valenced emotions.

Mu rhythm suppression is a widely adopted measure of sensorimotor activation [52,53]. Mu suppression (8–13 Hz in adults) manifests as electroencephalographic amplitudes that are below baseline (i.e., resting state) values during action execution, action observation, and imitation. Thus, mu suppression can be used as an index of mirror neuron system activation [54] and/or a neural signature of empathy. Researchers reported mu suppression as a response to the observation of emotional expressions [6], perception of pain in others [55,56], and trait empathy [57]. Other researchers reported mu suppression as a response to the adopting other individuals' perspectives [58] and inferring the emotional state of others [3]. These studies suggest that mu suppression may be engaged in emotional and cognitive empathic processes: experience sharing and mentalizing. Nevertheless, whether mu suppression reflects sensorimotor activity or empathy has been questioned [53,59]. It is important to distinguish the “mu activity” of the central electrodes from the potential leakage of “alpha activity” of the occipital region. The central mu activity is considered an individual's sensorimotor resonance, leading to empathy, while the occipital alpha activity involves visual or attentional processing [59,60]. Therefore, the present study used both the sensor-level mu suppression and the source-level sensorimotor activity as neural correlates to depict the effect of empathizer response on one's own empathic responses.

In this study, our primary objective was to investigate how empathizer feedback influences empathic responses to positive and negative emotions in naturalistic social situations. To maximize ecological validity, we designed a novel paradigm involving communications related to emotionally charged topics. Considering that auditory information is crucial to the process of mentalizing [61] and that the existence of visual information would increase the complexity of empathic processing [62], we adopted an audio-only paradigm: dyad members of this study were seated face-to-face with a plastic board placed between them to prevent them from seeing each other. We aimed to explore the effect of empathizer feedback on one's own empathic responses to positive emotion (happiness) and negative emotion (sadness) and its underlying neural correlates. Considering the complex interplay between emotional valence and components of empathy, we adopted three behavioral indicators of empathy (EA, EC, and EI) to examine the effects of feedback on cognitive and emotional empathy for happiness and sadness. Giving feedback may require top-down control (e.g., attention and working memory) for formulating appropriate responses (e.g., what to say and when) during a conversation. Meanwhile, it is also possible that not giving feedback requires top-down control for inhibiting spontaneous listener responses (e.g., “hmmm” and nodding). High cognitive load, which stems from top-down control, may reduce empathizers' cognitive empathy [4]. Therefore, the present study examined whether providing feedback or not providing feedback would decrease behavioral empathy scores and the activation of neural correlates related to empathetic responses (mu suppression and sensorimotor activity).

## 2 Materials and methods

### 2.1 Participants

We recruited 20 dyads of friends (19 males; mean age = 23.18 years, SD = 3.26, range = 20–34) who were primarily undergraduate or graduate students through advertisements on the university campus. The inclusion criteria included right-handedness, normal or corrected-to-normal vision and hearing, and no prior psychiatric or neurological illness. Depression has been linked to diminished empathy [63] and negative bias [64], which individuals selectively perceive, focus on, remember, or express negative experiences. Therefore, three of the dyads were excluded from the analysis due to Beck Depression Inventory (BDI-IA) scores indicating moderate depression, resulting in a final sample of 17 dyads (14 males; mean age = 23.44 years, SD = 3.42, range = 20–34). All participants provided written informed consent and received monetary compensation for participating in this study. All procedures were explained to the participants before the experiment and were conducted in accordance with approved guidelines. The study was approved by the Research Ethics Committee for Human Participant Protection of National Chiao Tung University (NCTU-REC-108-020E).

### 2.2 Stimuli

#### 2.2.1 Questionnaires

The Empathy Quotient (EQ) was used to measure empathy, including cognitive empathy and emotional reactivity [65]. The Beck Depression Inventory (BDI-IA) [66] was used to assess the emotional state of participants. The Friendship Inventory [67] was used to assess the relationships of participants.

#### 2.2.2 Written materials

To keep the participants on track during the speaking/listening stage of the experiment, they were permitted to check the keywords that they had written prior to the experiment. They were first asked to recall 10 happy and 10 sad life events that they had previously experienced without specifying that these events should involve the dyad or be solely individual experiences. This approach was intended to simulate a naturalistic range of emotional storytelling in participants’ real-life experiences. They were then provided with two wordlists of everyday items (e.g., soap) which were collected from Extended HowNet 2.0 [68]. Finally, they were asked to compile a list of keywords representative of each event (Happy and Sad conditions) and each item (Neutral condition).

#### 2.2.3 Music clips

**Fig. 1:**
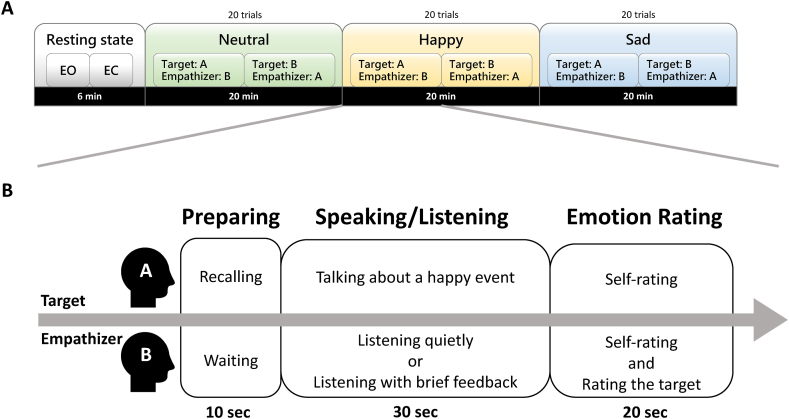
Procedure. (A) Overview of the resting and communication sessions consisting of Neutral, Happy, and Sad conditions; (B) Task flow of each trial. The target and empathizer were asked to listen to music clips followed by the speaking/listening stage and emotion rating stage. Note: EO, eyes-open; EC, eyes-closed; A, participant A; B, participant B.

To help the target recall emotional events in a congruent mood [69,70], 10 distinctly happy music clips and 10 distinctly sad music clips were played during the Happy and Sad conditions, respectively. Likewise, 10 distinctly neutral music clips were used to elicit a neutral emotional state. To instill a sense of emotional neutrality in empathizers, one distinctly neutral music clip (randomly selected from a collection of 30 clips) was played while waiting period (Fig. 1). The happy and sad music clips were collected from a set of 110 film music excerpts [71], whereas the neutral music clips were collected from ZapSplat (www.zapsplat.com/). The length of each music clip was 10 s.

### 2.3 Task

The study comprised two visits in which empathizers listened to targets with no feedback or little feedback. On average, there was a 24.41-day interval between the visits. At the beginning of each visit, the participants completed questionnaires. The experiment consisted of one resting session and one communication session (Fig. 1). During the resting session, EEG recordings were collected under 3-min eyes-closed and 3-min eyes-open resting-state conditions. A blocked design was employed to examine the effects of emotions. The communication session began with the Neutral condition followed by the Happy and Sad conditions. Such design kept the Neutral condition free of any potential sustained effect of emotion caused by the Happy or Sad conditions [37,72]. Note that the order of the Happy and Sad conditions was counterbalanced across dyads (e.g., Dyad 1: Neutral-Happy-Sad, Dyad 2: Neutral-Sad-Happy, etc.).

Each emotion condition consisted of 20 trials. For each emotion condition, in the initial 10 trials, one member of the dyad (e.g., participant B) was designated as the empathizer, while the other (e.g., participant A) was assigned as the target. Following the first 10 trials in that condition, the participants switched roles for the subsequent ten trials (e.g., participant A as the empathizer and participant B as the target). Each trial began with a 10-sec preparing stage, during which the target viewed the written materials (see the “Written materials” section) and recalled a happy/sad event or imagined how to describe an object while simultaneously listening to a mood-inducing music clip. At the same time, the empathizer was asked to wait while listening to a neutral music clip. In the subsequent 30-sec speaking/listening stage, the target viewed the written materials while verbally describing an emotional life event (happy or sad) or an everyday item (neutral). At the same time, the empathizer listened attentively to the target without providing feedback (i.e., UNABLE to respond) or with brief feedback, such as “hmmm” or “wow” (i.e., ABLE to respond). Finally, in the emotion rating stage, the target rated one's level of arousal (i.e., self-reported measure of emotional intensity), which was used to verify the emotional intensity of the reported stories and served as the reference of EA and EC. The empathizer rated both one's and the target's arousal levels on a scale from 0 (Not at all) to 10 (Extremely).

**Fig. 2:**
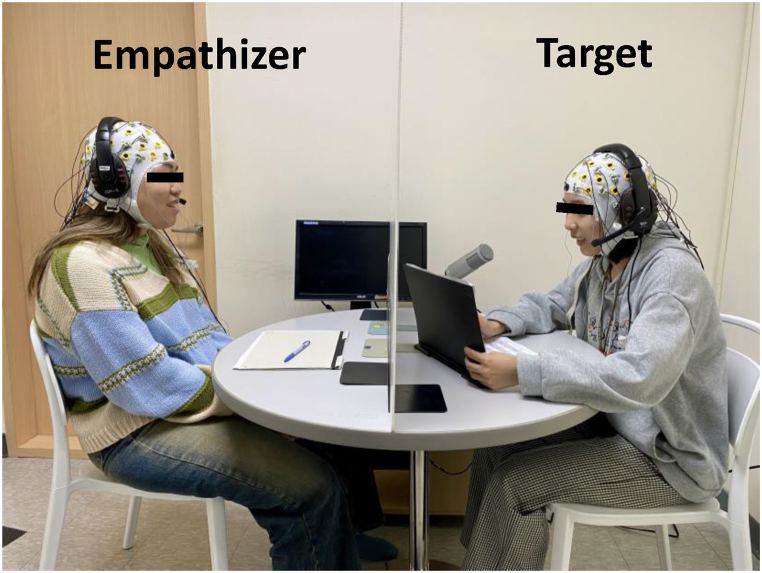
Experiment setting in which the participant pairs sat face-to-face with a plastic board between them to obscure their view.

The experiment was conducted in a soundproof laboratory. To prevent participants from seeing each other, dyad members sat face-to-face with a plastic board placed between them (Fig. 2). During the experiment, participants were given prerecorded instructions via headphones. The vocal responses of the target and the empathizer were recorded for further analysis. Every vocal response was considered feedback, including laughing, particles (e.g., “hmmm”), and complete/incomplete sentences.

### 2.4 EEG data acquisition

EEG data were recorded using two 32-channel elastic caps based on the extended international 10–20 system using the BrainVision actiCAP system (https://brainvision.com/). Data were referenced to FCz with the ground electrode placed at FPz. Impedance was maintained below 10 kΩ. EEG data were recorded using a 0.05–100 Hz band-pass filter at a sampling rate of 1,000 Hz. Electrooculographic (EOG) and electrocardiogram (ECG) signals were recorded respectively to monitor eye blinks and heart rate.

### 2.5 EEG data processing

EEG data recorded during the speaking/listening (1–29 s) and eyes-open resting-state (3–183 s) stages were analyzed using the EEGLAB software [73]. Raw EEG data were resampled to 500 Hz, re-referenced to the average of all electrodes using the Reference Electrode Standardization Technique [74], and band-pass filtered to 0.5–50 Hz. Artifacts resulting from eye blinks and movements were removed using Artifact Subspace Reconstruction (ASR) [75], Independent Component Analysis (ICA) [76], and ICLabel toolbox [77]. After data preprocessing, the power in the 8–13 Hz range (i.e., mu/alpha rhythm, see the “EEG measures”) of six electrodes (C3, Cz, C4, O1, Oz, and O2) was obtained using a Fast Fourier Transform (FFT) at 0.25 Hz intervals with a 4-sec Hanning window and 50 % overlap.

### 2.6 Neural correlates

A mu suppression/enhancement index was defined as a log ratio of the mean spectral power in the central region (C3, Cz, and C4) during the speaking/listening stage (happy, sad) relative to the mean spectral power during the eyes-open resting stage (baseline). Due to the inherently non-normal distribution of the original ratio scores, a log transformation was applied [3,57,78,79]. A higher negative mu suppression index indicated more pronounced mu suppression, suggesting higher sensorimotor cortical activation.

An alpha suppression/enhancement index was defined as a log ratio of the spectral power in the occipital region (O1, Oz, and O2) during the speaking/listening stage (happy, sad) relative to the mean spectral power during the baseline (resting-state) stage. The alpha suppression index was used to ensure that the effect of mu suppression was specific to the central region.

**Fig. 3:**
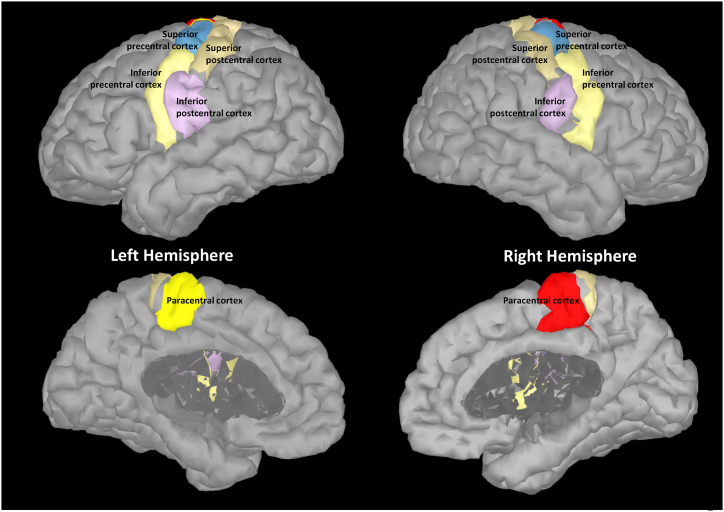
Selected scouts of sensorimotor regions in source-level analysis.

To estimate the activation of the sensorimotor cortex, we utilized the standardized LORETA (sLORETA) source reconstruction [80] as implemented in the MATLAB toolbox Brainstorm [81]. The head model was computed using individual brain anatomies (Siemens MAGNETOM Prisma MRI scanner). The source activation was obtained by projecting individual activation into the USCBrain template [82]. Our regions of interest (ROIs) consisted of ten sensorimotor areas, including the bilateral paracentral cortex, superior/inferior precentral cortex, and superior/inferior postcentral cortex (Fig. 3). After extracting time series for each participant, the alpha power of each ROI was obtained using the same procedure as in the aforementioned sensor-level analysis.

### 2.7 Behavioral measures

Three dependent variables served as indicators of empathy. The first indicator was empathic accuracy (EA), which indicates the accuracy with which the empathizer judged the emotional state of the target. Previous researchers have measured empathic accuracy by correlating the inferences of the empathizer with the self-reports of the target [3,83,84]. To avoid possible interference (behavioral or EEG) due to continuously providing ratings, the empathizers and targets were asked to provide ratings only once at the end of each trial. Note that our use of only two scores precluded the use of correlation analysis; therefore, we calculated empathic accuracy of each trial as follows [85]:

EA = 10 - | *inference*_*em*_ - *self*_*tar*_ |

where *inference*_*em*_ indicates the empathizer inference score and *self*_*tar*_ indicates the target self-report score. *self*_*tar*_ served as the baseline in determining the level of accuracy. Note that absolute values were used here, due to the fact that the direction of empathic accuracy (i.e., underestimation or overestimation) was not the focus of this study. For example, if the empathizer inference score was 8 and the target self-report score was 8, then the EA score would be 10. If the empathizer inference score was 8 and the target self-report score was 2, then the EA score would be 4. The final EA scores were the mean of 10 trials.

The second indicator was emotional contagion (EC), which indicates how closely the empathizer resonated with the emotional state of the target. Emotional contagion was calculated as follows:

EC = 10 - | *self*_*em*_ - *self*_*tar*_ |

where *self*_*em*_ indicates the empathizer self-report score and *self*_*tar*_ indicates the target self-report score. *self*_*tar*_ served as the baseline in determining the level of contagion. Again, absolute values were used here. Note that the emotional contagion score was proportional to the strength with which the empathizer resonated. The final EC scores were the mean of 10 trials.

The third indicator was emotional intensity (EI), which was obtained from the empathizer's self-rated arousal levels (i.e., *self*_*em*_*)*. The emotional intensity score was proportional to the level of intensity of the empathizer experienced [40,49]. The final EI scores were the mean of 10 trials.

### 2.8 Statistical analysis

The statistical analysis was conducted using IBM SPSS 22.0. To examine the effect of emotion induction of the proposed experimental paradigm, a one-way (emotion: happy, sad, neutral) repeated measures ANOVA was conducted, using the emotional intensity of the target as the dependent variable. To examine the effect of emotion on the mean number of empathizer feedback per trial, a one-way (emotion: happy, sad, neutral) repeated measures ANOVA was conducted. The effects of feedback and positive/negative emotion on empathy during naturalistic communication were assessed by conducting 2 (feedback: unable, able) × 2 (emotion: happy, sad) repeated measures ANOVAs on the behavioral measures (EA, EC, and EI) and EEG measures (suppression index of six electrodes and alpha activation of ten sensorimotor areas) with Bonferroni correction (*P* < 0.05). Paired-samples t-tests were used as planned contrasts to determine whether the above measures differed (1) between emotion conditions based on each feedback condition and (2) between feedback conditions based on each emotion condition with Bonferroni correction (*P* < 0.05). Pearson correlation analysis was used to examine the relationship between behavioral measures (EA, EC, EI, and EQ scores) and EEG measures with Bonferroni correction (*P* < 0.05). Mu suppression difference was further estimated by subtracting mu suppression under the Neutral condition from that under the Happy/Sad condition, followed by Pearson correlation analysis examining the relationship between empathy scores and mu suppression difference.

To elucidate whether the effects of feedback and positive/negative emotion function differently in the central and occipital regions, we examined the effects of feedback, positive/negative emotion, and location on the alpha suppression/enhancement index associated with the central electrodes using a 2 (feedback: unable, able) × 2 (emotion: happy, sad) × 2 (location: central, occipital) repeated measure ANOVA on the EEG measures (mean suppression/enhancement index of three electrodes from each region) with Bonferroni correction (*P* < 0.05).

## 3 Results

### 3.1 Behavioral results

#### 3.1.1 Emotional intensity of the target

**Fig. 4:**
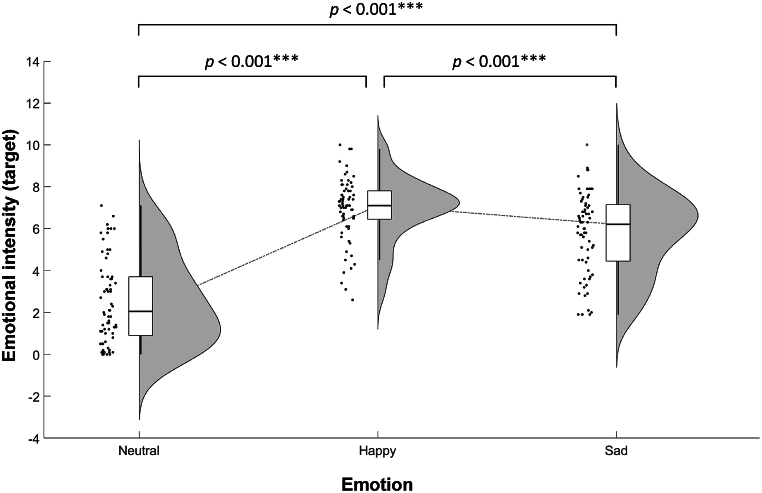
Emotional intensity of the target, where higher values indicate higher arousal.

Using a one-way (emotion: happy, sad, neutral) repeated measures ANOVA, we examined the effect of emotion induction of the proposed experimental paradigm. We observed significant effects of emotion (*F* (2, 66) = 124.177, *p* < 0.001, *η*^*2*^_*p*_ = 0.79) (Fig. 4). Post-hoc analysis revealed that emotional intensity of the target under the Happy condition was significantly higher than under the Sad (*p* < 0.001) and Neutral (*p* < 0.001) conditions. Emotional intensity under the Sad condition was significantly higher than under the Neutral condition (*p* < 0.001). These results are an indication that the proposed experimental paradigm was successful in inducing high-intensity emotions in the target, particularly under the Happy condition.

#### 3.1.2 Empathizer feedback

**Fig. 5:**
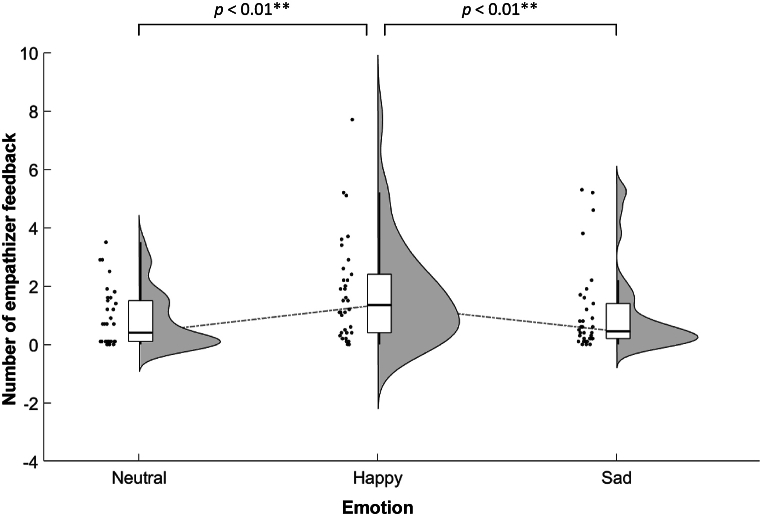
The mean number of empathizer feedback per trial.

Using a one-way (emotion: happy, sad, neutral) repeated measures ANOVA, we examined the effect of emotion on the mean number of empathizer feedback per trial. We observed significant effects of emotion (*F* (2, 66) = 8.261, *p* < 0.001, *η*^*2*^_*p*_ = 0.20). Post-hoc analysis revealed that the number of empathizer feedback under the Happy condition was significantly higher than under the Sad (*p* = 0.004) and Neutral (*p* = 0.006) conditions (Fig. 5). We observed no significant difference between the Sad and Neutral conditions (*p* = 0.992). These results provided an indication that the targets responded more frequently under the Happy condition.

#### 3.1.3 Empathic accuracy

**Fig. 6:**
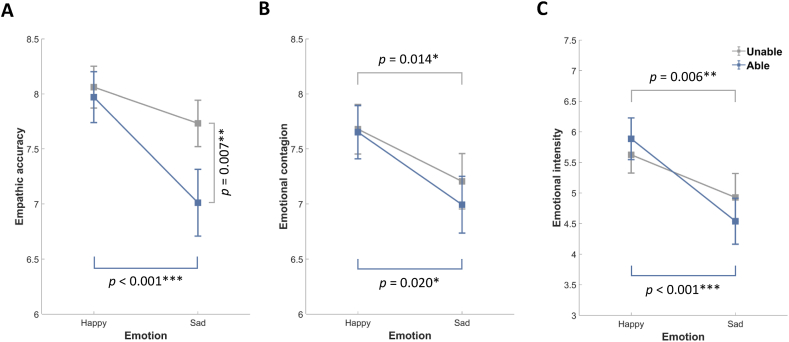
Behavioral results: (A) Empathic accuracy, where higher values indicate the accuracy with which the empathizer characterized the emotional state of the target; (B) Emotional contagion, where higher values indicate the closeness with which the empathizer resonated with the emotional state of the target. (C) Emotional intensity, where higher values indicate higher arousal. Data points indicate the mean (±SE) value for a given (color-coded) feedback condition. (For interpretation of the references to color in this figure legend, the reader is referred to the Web version of this article.)

Fig. 6A presents the effects of feedback and positive/negative emotion on EA using 2 (feedback: unable, able) × 2 (emotion: happy, sad) repeated measures ANOVA. We observed a significant main effect of feedback (*F* (1, 33) = 6.279, *p* = 0.017, *η*^*2*^_*p*_ = 0.16) with higher EA scores under the Unable condition than under the Able condition. We also observed a significant main effect of emotion (*F* (1, 33) = 15.196, *p* < 0.001, *η*^*2*^_*p*_ = 0.32) with higher EA scores under the Happy condition than under the Sad condition and a significant interaction between feedback and emotion (*F* (1, 33) = 4.698, *p* = 0.038, *η*^*2*^_*p*_ = 0.12). Post-hoc analysis revealed higher EA scores under the Happy-Able condition than under the Sad-Able condition (*p* < 0.001) with no significant difference between the Happy-Unable and Sad-Unable conditions (*p* = 0.106). Post-hoc analysis also revealed higher EA scores under the Sad-Unable condition than under the Sad-Able condition (*p* = 0.007) with no significant difference between the Happy-Unable and Happy-Able conditions (*p* = 0.609). Taken together, these results suggest that accurately inferring sadness is more difficult than accurately inferring happiness and that remaining silent is helpful in inferring emotions (particularly sadness). We examined the relationship between the emotional intensity of the target and the EA scores of the empathizer and observed no significant correlation with Bonferroni correction.

#### 3.1.4 Emotional contagion

Fig. 6B presents the effects of feedback and positive/negative emotion on EC using 2 (feedback: unable, able) × 2 (emotion: happy, sad) repeated measures ANOVA. We observed a significant main effect of emotion (*F* (1, 33) = 13.302, *p* = 0.001, *η*^*2*^_*p*_ = 0.29), and post-hoc analysis revealed significantly higher EC scores under the Happy condition than under the Sad condition. Note that the results of post-hoc analysis were consistent with those of planned contrasts used to determine whether EC scores differed between Happy and Sad conditions as a function of Unable (*p* = 0.014) and Able (*p* = 0.020) conditions. We observed no significant main effect of feedback (*F* (1, 33) = 0.604, *p* = 0.442, *η*^*2*^_*p*_ = 0.02) nor any interaction between emotion and feedback (*F* (1, 33) = 0.293, *p* = 0.592, *η*^*2*^_*p*_ = 0.01). Taken together, these results suggest that sadness is less contagious than is happiness and that feedback does not significantly contribute to emotional contagion. We examined the relationship between the emotional intensity of the target and the EC scores of the empathizer. Correlation analysis revealed significant positive correlations under the Happy conditions (Happy-Unable: *r* = 0.471, *p* = 0.020; Happy-Able: *r* = 0.440, *p* = 0.037) and the Sad-Able condition (*r* = 0.469, *p* = 0.020) with Bonferroni correction.

#### 3.1.5 Emotional intensity of the empathizer

Fig. 6C presents the effects of feedback and positive/negative emotion on EI using 2 (feedback: unable, able) × 2 (emotion: happy, sad) repeated measures ANOVA. We observed a significant main effect of emotion (*F* (1, 33) = 32.538, *p* < 0.001, *η*^*2*^_*p*_ = 0.50), and post-hoc analysis revealed significantly higher EI scores under the Happy condition than under the Sad condition. Note that the results of post-hoc analysis were consistent with those of planned contrasts used to determine whether EI scores differed between Happy and Sad conditions as a function of Unable (*P* = 0.006) and Able (*P* < 0.001) conditions. We observed no significant main effect of feedback (*F* (1, 33) = 0.117, *p* = 0.735, *η*^*2*^_*p*_ < 0.01) nor any interaction between emotion and feedback (*F* (1, 33) = 2.893, *p* = 0.098, *η*^*2*^_*p*_ = 0.08). These results suggest that sadness elicits less arousing than happiness and that feedback does not significantly contribute to emotional intensity. We examined the relationship between the emotional intensity of the target and the EC scores of the empathizer. Correlation analysis revealed significant positive correlations under all four conditions (Happy-Unable: *r* = 0.653, *p* < 0.001; Happy-Able: *r* = 0.696, *p* < 0.001; Sad-Unable: *r* = 0.722, *p* < 0.001; Sad-Able: *r* = 0.687, *p* < 0.001) with Bonferroni correction.

### 3.2 Results of neural correlates

#### 3.2.1 Mu suppression at centrode electrodes

**Fig. 7:**
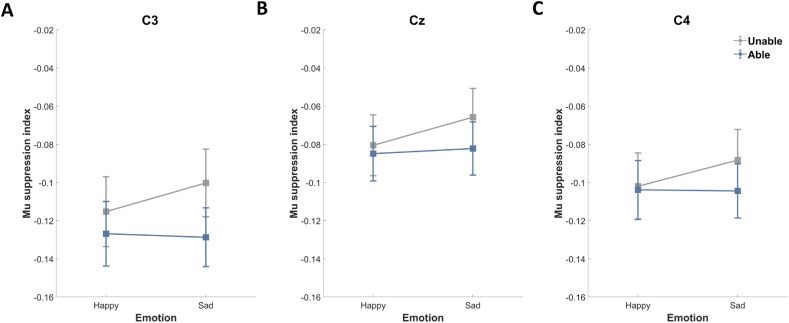
Mu suppression index at (A) C3, (B) Cz, and (C) C4. More negative values indicate greater mu suppression (i.e., higher sensorimotor cortical activation). Data points indicate the mean (±SE) value for a given (color-coded) feedback condition. (For interpretation of the references to color in this figure legend, the reader is referred to the Web version of this article.)

In interpreting data from the central electrodes (Fig. 7), we first examined whether the mu suppression index differed from 0 using one-sample t-tests. All three central electrodes indicated significant suppression (*ps* < 0.001).

We then examined the effects of feedback and positive/negative emotion on the mu suppression index associated with the central electrodes using 2 (feedback: unable, able) × 2 (emotion: happy, sad) repeated measures ANOVA. We observed no significant main effect or interaction. Our discovery of a significant interaction between emotion and feedback in the behavioral results (see the **“**Empathic accuracy” section) prompted further analysis involving two planned contrasts using paired-sample t-tests. The first planned contrast was meant to determine whether mu suppression differed between Happy and Sad conditions based on each feedback condition. We observed no significant difference between the Happy-Unable and Sad-Unable conditions, nor between the Happy-Able and Sad-Able conditions.

The second planned contrast aimed to investigate whether mu suppression varied between the Unable and Able conditions across different emotional states. While we noticed a tendency for stronger activation in response to feedback when the empathizer was experiencing sadness, we did not find any significant differences between the Happy-Unable and Happy-Able conditions, nor between the Sad-Unable and Sad-Able conditions.

#### 3.2.2 Correlation between behavioral measures and mu suppression

We examined the relationship between behavioral measures (EA, EC, EI, and subscales of EQ) and mu suppression. Correlation analysis revealed no significant correlation between mu suppression and EA, EC, nor EI with Bonferroni correction. To further illuminate the association between sensorimotor mu activity and empathy, we also examined the relationship between behavioral measures and mu suppression difference. Correlation analysis revealed significant positive correlations between EC and mu suppression difference at C4 under the Sad-Able condition (*r* = 0.501, *p* = 0.030). Correlation analysis revealed significant positive correlations between cognitive empathy (a subscale of EQ) and mu suppression at Cz under the Sad-Unable condition (*r* = 0.495, *p* = 0.035). These results suggest that under the negative emotion condition, empathizers with low cognitive empathy exhibit high mu suppression in the sensorimotor regions only while listening non-reactively.

#### 3.2.3 Alpha power of sensorimotor cortical areas

We examined the effects of feedback and positive/negative emotion on the alpha power of the selected sensorimotor ROIs using 2 (feedback: unable, able) × 2 (emotion: happy, sad) repeated measures ANOVA. We found a significant main effect of emotion with higher alpha power under the Happy condition than under the Sad condition in eight sensorimotor areas (see Table 1). We observed no significant main effect of feedback or interaction.

**Table 1:** Results of two-way repeated measures ANOVA of alpha activation of sensorimotor areas using feedback and emotion as within-subject factors.

**Region**	Feedback		Emotion		Feedback × Emotion	
	F-value	p-value	F-value	p-value	F-value	p-value
**Left Hemisphere**						
Paracentral cortex	0.018	>0.999	15.812	**0.004∗∗**	1.851	>0.999
Superior precentral cortex	0.143	>0.999	12.131	**0.014∗**	1.408	>0.999
Inferior precentral cortex	1.458	>0.999	26.499	**<0.001∗∗∗**	2.209	>0.999
Superior postcentral cortex	0.053	>0.999	18.356	**0.001∗∗**	1.950	>0.999
Inferior postcentral cortex	2.316	>0.999	24.516	**<0.001∗∗∗**	2.981	0.936
**Right Hemisphere**						
Paracentral cortex	0.058	>0.999	7.193	0.113	1.649	>0.999
Superior precentral cortex	0.853	>0.999	7.189	0.114	1.992	>0.999
Inferior precentral cortex	1.589	>0.999	13.603	**0.008∗∗**	0.528	>0.999
Superior postcentral cortex	0.006	>0.999	17.222	**0.002∗∗**	3.047	0.902
Inferior postcentral cortex	1.363	>0.999	16.066	**0.003∗∗**	1.307	>0.999

Note: ∗*p* < 0.05, ∗∗*p* < 0.01, ∗∗∗*p* < 0.001 (corrected for multiple comparisons).

Post-hoc analysis revealed higher alpha power under the Happy-Unable condition than under the Sad-Unable condition in seven sensorimotor areas (see Table 2), such as bilateral paracentral cortex and postcentral cortex, with no significant difference between the Happy-Able and Sad-Able conditions. Post-hoc analysis revealed no significant difference between Happy-Unable and Happy-Able conditions nor between Sad-Unable and Sad-Able conditions with Bonferroni correction. These source-level results suggest that in the sensorimotor regions, the power induced by sadness is less pronounced when the empathizer is not providing feedback, which is consistent with the sensor-level results. Correlation analysis revealed no significant correlation between the alpha power of sensorimotor areas and empathy scores (EA, EC, and EI).

**Table 2:** Results of paired-samples t-tests.

**Region**	Happy-Sad			
	Unable		Able	
	t-value	p-value	t-value	p-value
**Left Hemisphere**				
Paracentral cortex	3.831	**0.005∗∗**	1.023	>0.999
Superior precentral cortex	2.539	0.160	0.881	>0.999
Inferior precentral cortex	4.166	**0.002∗∗**	1.531	>0.999
Superior postcentral cortex	3.163	**0.033∗**	1.237	>0.999
Inferior postcentral cortex	4.372	**0.001∗∗**	1.735	0.921
**Right Hemisphere**				
Paracentral cortex	3.349	**0.020∗**	0.531	>0.999
Superior precentral cortex	2.782	0.089	0.598	>0.999
Inferior precentral cortex	2.333	0.259	2.351	0.248
Superior postcentral cortex	4.137	**0.002∗∗**	1.826	0.769
Inferior postcentral cortex	2.931	0.061	1.802	0.807

Note: ∗*p* < 0.05, ∗∗*p* < 0.01 (corrected for multiple comparisons).

#### 3.2.4 Alpha enhancement at occipital electrodes

We examined the effects of feedback, positive/negative emotion, and location on the alpha suppression/enhancement index associated with the electrodes using a 2 (feedback: unable, able) × 2 (emotion: happy, sad) × 2 (location: central, occipital) repeated measure ANOVA. The results revealed a significant effect of location (*F* (1, 33) = 55.776, *p* < 0.001, *η*^*2*^_*p*_ = 0.628), a significant interaction between location and feedback (*F* (1, 33) = 7.349, *p* = 0.011, *η*^*2*^_*p*_ = 0.182), a significant interaction between location and emotion (*F* (1, 33) = 8.671, *p* = 0.006, *η*^*2*^_*p*_ = 0.208), and a significant interaction between feedback and emotion (*F* (1, 33) = 4.143, *p* = 0.050, *η*^*2*^_*p*_ = 0.112). These results indicate that the effects of feedback and positive/negative emotion functioned differently in the central and occipital regions.

**Fig. 8:**
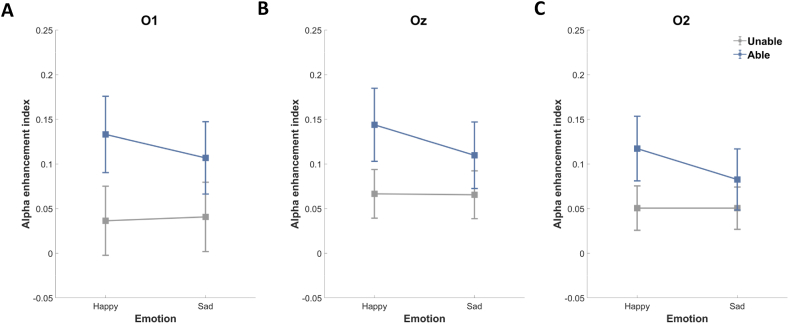
Alpha enhancement index at (A) O1, (B) Oz, and (C) O2. More positive values indicate greater alpha enhancement (i.e., lower cortical activation). Data points are color-coded to indicate the feedback condition. (For interpretation of the references to color in this figure legend, the reader is referred to the Web version of this article.)

In the occipital electrodes (Fig. 8), alpha activity revealed enhancement rather than suppression under the Happy-Able condition. All three occipital electrodes presented significant enhancement (*ps* < 0.05) in one-sample t-tests (differing from 0) with Bonferroni correction. These results are indicative of reduced cortical activation (i.e., high alpha enhancement), which was more pronounced when the empathizers were providing feedback to positive narratives.

To establish that mu suppression effects were specific to the central region, we examined the effects of feedback and positive/negative emotion on the alpha enhancement index at the occipital electrodes using 2 (feedback: unable, able) × 2 (emotion: happy, sad) repeated measures ANOVA. We observed no significant main effect or interaction with Bonferroni correction.

The significant interaction effect between feedback and emotion on behavioral results prompted further analysis. Planned contrasts were used to determine whether alpha enhancement differed between Happy and Sad conditions as a function of feedback condition. Alpha enhancement was significantly more pronounced under the Happy-Able condition than under the Sad-Able condition at O2 (*p* = 0.045) with Bonferroni correction. We observed no significant difference in alpha enhancement between Happy-Unable and Sad-Unable conditions. These results suggest that sadness-induced cortical activation in the right occipital regions was more pronounced when the empathizers were providing feedback.

Planned contrasts were also used to determine whether alpha enhancement differed between Unable and Able conditions as a function of emotion condition. No significant difference was observed. Finally, we examined the relationship between behavioral measures (EC, EA, EI, and subscales of EQ) and alpha enhancement. No significant correlation was observed between behavioral measures and alpha enhancement, which suggests that alpha enhancement is not related to empathy.

#### 3.2.5 Friendship inventory

No significant correlation was observed between friendship scores and EA, EC, EI.

## 4 Discussion

In the real world, empathy involves dynamic interactions between an empathizer and a target. Most previous research on this topic involved the empathizer passively receiving emotional information without providing any feedback [[3], [4], [5], [6]]. To our knowledge, our study is the first to explore the effects of empathizer feedback on empathic responses to positive and negative emotions among friends engaging in naturalistic communication. Empathizer feedback was shown to decrease EA, only when the empathizer received sad narratives, but without effect on EC or EI. Participants also had greater difficulty assessing sadness than they did happiness, particularly when the empathizer provided feedback. Empathizers presented lower alpha activity in the sensorimotor cortical areas only while receiving sad narratives without providing feedback. Our findings demonstrate that only empathy for sadness is affected by feedback, while empathy for happiness remains unaffected. Additionally, our findings indicate that giving feedback diminishes cognitive empathy but not emotional empathy.

Empathy is a complex, multifaceted behavior involving cognitive and emotional processes [47]. Researchers have reported that empathy can be influenced by cognitive loads, such as attention and working memory [4,[86], [87], [88]]. Although our study did not manipulate the level of cognitive load, we employed three indicators of empathy (EA, EC, and EI), which involve different levels of cognitive mentalizing, to elucidate the effect of empathizer feedback on cognitive and emotional empathy. Our behavioral results revealed a tendency for empathizer feedback to suppress EA (an indicator of cognitive processes) rather than EC nor EI (indicators of emotional process). Our findings are consistent with previous studies that reported that a high cognitive load can reduce one's capacity for top-down mentalizing and caregiving intention while the automatic/bottom-up emotional component of empathy remains unaffected [4,[86], [87], [88]]. Specifically, a high cognitive load (e.g., memorizing an 8-digit number when viewing emotion-inducing images) was shown to decrease self-reported intention to provide care [89]. Morelli and Lieberman [4] found that a high cognitive load decreased subjective empathy and neural activities related to cognitive empathy (dorsal medial prefrontal cortex, medial prefrontal cortex, temporoparietal junction), while neural activities related to emotional empathy (anterior insula and septal area) were not influenced. We speculate that participants in the Able condition require more cognitive resources than did those in the Unable condition to determine when and how to respond appropriately. We further posit that assigning cognitive resources to feedback recruits high-level mentalizing/perspective-taking rather than low-level experience sharing. This may explain why only EA (pertaining to high-level cognitive empathy/mentalizing) was lower among participants in the Able condition. Notably, our findings are inconsistent with previous reports [49,90]. Bajouk and Hansenne [90] reported that cognitive load does not affect empathy unless the scores of the Interpersonal Reactivity Index were considered. Tremblay et al. [49] reported that cognitive load increases men's empathy. These inconsistent findings may result from individual differences, such as trait empathy and gender. Thus, it is important for future studies to consider individual difference factors that may influence empathy.

We examined the nature of empathic responses to happiness and sadness with the aim of elucidating the effects of empathizer feedback on empathic responses to positive and negative emotions. We found that EA, EC, and EI levels were lower under the Sad condition than under the Happy condition. We also found that alpha activity of sensorimotor cortical areas was lower under the Sad condition than the Happy condition, which suggests that the sensorimotor activity induced by sadness was less than that induced by happiness. Previous studies have demonstrated that automatic mimicry/emotion sharing involves sensorimotor activity [1,91]. Our source-level findings suggest that sadness impeded spontaneous emotion sharing. Considering the neural and behavioral evidence obtained in this study, it appears that sadness is less contagious and harder to judge accurately than is happiness. There are two possible explanations for this. The first explanation of asymmetry in empathic responses to positive and negative emotions is that positive material is associated with approach behavior whereas negative information is associated with avoidance [92]. Accordingly, individuals would be more willing to engage in positive conversations, which is consistent with our finding that the targets responded more frequently under the Happy condition.

The second explanation is that the intensity of emotions experienced by the target differs as a function of emotional valence (i.e., positive vs. negative). In the current study, the targets self-reported that the emotional intensity under the Sad condition was lower than under the Happy condition, which suggests that their intention to express negativity was lower. Zaki et al. [83] claimed that expressivity and the production of emotional cues could influence EA levels. They found that when only auditory information was available for empathizers, targets with higher expressivity tended to display more positive verbal cues and corresponding higher levels of EA. We therefore speculate that the low EA for sadness in the current study can be attributed to the low expressivity associated with negative emotions. Furthermore, we found that the intensity of spoken stories only positively correlated with EC and EI; no significant correlation was found with EA. Such findings imply that the intensity of emotions influences empathy's cognitive and emotional components differently.

The sensorimotor cortex plays a key role in empathy [1]. Researchers have previously reported that the right lateralization of mu suppression was correlated with empathic capacity [56,79,84]. In the current study, the source-level results show no significant interaction between feedback and emotion with multiple-comparison corrections. However, our planned contrasts reveal that in the sensorimotor regions, the power induced by sadness is less pronounced when the empathizer is not providing feedback, with no significant difference between the Happy-Able and Sad-Able conditions. Such results may provide support that sensorimotor activation is associated with empathy and emotion. Due to the fact that the processing of negative emotions and providing feedback are both highly demanding in terms of top-down cognitive load, we speculate that the reallocation of cognitive resources may be the reason why the emotion effect occurs only under the Unable condition. In future investigations, it might be possible to include more brain regions related to empathy and more advanced methods, such as functional connectivity analysis, to clarify the neural mechanisms of empathy in a complex naturalistic social environment.

In the real world, appropriate feedback from an empathizer is crucial to the smooth flow of social interactions [8]. Feedback also requires top-down control, (e.g., attention and working memory) to formulate appropriate responses (e.g., what to say and when) during a conversation. The novel experimental paradigm in the current study helped to elucidate the effects of additional cognitive load (due to the provision of feedback) on the processing of sadness. The EA of empathizers was lower under the Sad-Able condition than under the Sad-Unable condition, while no difference was observed between happiness-associated conditions (Happy-Able vs. Happy-Unable). These results suggest that empathizer feedback hampered the empathic process in response to sadness but not in response to happiness. We speculate that the additional load imposed by top-down control (e.g., providing feedback) decreased empathic responses in situations where high cognitive demand was required (e.g., processing sadness). In line with previous studies, we also determined that remaining silent is helpful in processing sadness [[93], [94], [95]], which suggests that foregoing the temptation to provide verbal feedback could be beneficial to the processing of negative emotions in the target. Note that in the current study, the speaking/listening stage (30 s) was shorter than in most normal conversations, such that the potential adverse effects of remaining silent were probably diminished. Our findings suggest that when dealing with a target suffering from sadness, brief silences could facilitate the emotional understanding of the empathizer. Nonetheless, the diversity and complexity of social interactions necessitate further research on the impact of empathizer feedback during unidirectionally versus bidirectionally naturalistic communication.

This study was subject to a number of limitations, which should be considered in the interpretation of our results. First, auditory information is crucial to the process of mentalizing, such that the absence of visual information could presumably diminish the processing involved in mirroring and experience sharing [61,96]. Second, our study design of real-time communication closely resembled a naturalistic setting; however, the efficacy of the manipulation of emotion and feedback could not be verified. In fact, some of the participants shared sad memories without any signs of inflection or even with a wry smile, thereby confusing the underlying tone of the communication, with a corresponding negative effect on the emotional impact of their words as well as the resulting empathic responses and feedback [62]. The relationships between empathizer feedback and cognitive load were not examined in this study, which remain unclear and need to be verified in the future. Third, the baseline used in this study was eyes-open resting state, instead of the neutral condition. In the early stage of behavioral data analysis, we observed that the EA and EC scores were higher under Neutral than Sad conditions (see Supplementary Fig. S1, Tables S1 and S2), indicating that empathic processing under Neutral condition was more pronounced than we had expected. This precluded using the Neutral condition as a baseline. Fourth, our sample size was small because of the difficulty of recruiting participants during the COVID-19 pandemic. Therefore, the same participants played both the target and empathizer, which may increase the complexity of the study. Moreover, in real-world situations, one person can be the target and empathizer in the same conversation. Future research could assess the impact of the role-playing factor. Fifth, we did not collect participants’ background information, such as socioeconomic status, which may influence their empathic responses. Future research should consider the potential impact of individual backgrounds and collect corresponding information.

Finally, we used mu suppression and alpha power as the sensorimotor cortical activation index, despite the fact that empathy correlates with the experience-sharing system (such as the premotor cortex, anterior cingulate cortex, and anterior insula) as well as the mentalizing system (such as the medial prefrontal cortex, precuneus, posterior cingulate cortex, and temporoparietal junction) [1,[97], [98], [99]]. The limitations mentioned above may reduce the effect size of mu suppression, resulting in insignificant results when multiple-comparison corrections are applied. Empathy in naturalistic social situations is complicated. Therefore, we recommend future studies on this topic include more real-life settings (i.e., visual information and back-and-forth conversation), more positive/negative emotion categories (i.e., contentment, anxiety, and anger), more evaluation (i.e., rating valence levels and continuous rating assessments), and more analysis methods (i.e., region-of-interest correlated with mentalizing system).

In summary, our research provides empirical evidence that understanding negative emotions is more challenging than understanding positive ones, particularly when providing feedback. Listening attentively and providing comfort is a common scenario for families dealing with patients or in general social interactions [[100], [101], [102]]. In clinical settings, psychotherapists often address their clients' concerns and offer support in their daily practice [103,104]. Our findings implicate the crucial roles of emotional valence and listener responses for empathy in social interactions. In real-world situations, empathizers frequently provide feedback in response to the story that they are hearing; however, there has been a dearth of research pertaining to the impact of feedback on empathic responses. This study contributes to the emerging research on the influence of empathizer feedback in naturalistic social settings. Empathizer feedback should be considered in future studies seeking to elucidate the neural mechanisms of empathy in a complex naturalistic social environment. We encourage future studies to broaden the scope of naturalistic social communication by engaging in back-and-forth conversations in multimodal environments, incorporating auditory and visual cues.

## Ethical statement

The study was approved by the Research Ethics Committee for Human participant Protection of National Chiao Tung University (NCTU-REC-108-020E). All procedures were explained to the participants before the experiment and were conducted in accordance with approved guidelines. All participants provided written informed consent and received monetary compensation for participating in this study.

## Data availability statement

The data that support the findings of this study are available on request from the corresponding author. The data are not publicly available due to privacy or ethical restrictions.

## Funding

This work was supported by the Ministry of Science and Technology in Taiwan (MOST) [108-2321-B-009-006-MY2 to Y.-S.C.; 111-2410-H-A49-049-MY3 to L.-F.C.] and Cheng Hsin General Hospital [CY11205 to L.-F.C.].

## CRediT authorship contribution statement

**Ruei-Jyun Hung:** Writing – review & editing, Writing – original draft, Visualization, Software, Project administration, Methodology, Investigation, Formal analysis, Conceptualization. **Intan Low:** Project administration, Investigation. **Hung-Chun Yeh:** Investigation, Data curation. **Po-Yu Wang:** Investigation, Data curation. **Yong-Sheng Chen:** Supervision, Resources, Funding acquisition, Conceptualization. **Li-Fen Chen:** Writing – review & editing, Writing – original draft, Visualization, Supervision, Resources, Methodology, Funding acquisition, Conceptualization.

## Declaration of competing interest

The authors declare that they have no known competing financial interests or personal relationships that could have appeared to influence the work reported in this paper.

## References

[1] de Waal F.B.M., Preston S.D. (Aug 2017). Mammalian empathy: behavioural manifestations and neural basis. Nat. Rev. Neurosci..

[2] Decety J., Meyer M. (2008). From emotion resonance to empathic understanding: a social developmental neuroscience account. Dev. Psychopathol..

[3] Genzer S., Ong D.C., Zaki J., Perry A. (Sep 1 2022). Mu rhythm suppression over sensorimotor regions is associated with greater empathic accuracy. Soc. Cognit. Affect Neurosci..

[4] Morelli S.A., Lieberman M.D. (2013). The role of automaticity and attention in neural processes underlying empathy for happiness, sadness, and anxiety. Front. Hum. Neurosci..

[5] Shahri F. (2024). I understand your pain but I do not feel it: lower affective empathy in response to others' social pain in narcissism. Front. Psychol..

[6] Moore A., Gorodnitsky I., Pineda J. (Jan 1 2012). EEG mu component responses to viewing emotional faces. Behav. Brain Res..

[7] Shamay-Tsoory S.G., Hertz U. (Jul 2022). Adaptive empathy: a model for learning empathic responses in response to feedback. Perspect. Psychol. Sci..

[8] Kluger A.N., Itzchakov G. (2022). The power of listening at work. Annual Review of Organizational Psychology and Organizational Behavior.

[9] Wang S., Lu J., Yu M., Wang X., Shangguan C. (Sep 25 2022). I'm listening, did it make any difference to your negative emotions?" Evidence from hyperscanning. Neurosci. Lett..

[10] Kawamichi H. (2015). Perceiving active listening activates the reward system and improves the impression of relevant experiences. Soc. Neurosci..

[11] Gamble R.S., Henry J.D., Decety J., Vanman E.J. (2024). The role of external factors in affect-sharing and their neural bases. Neurosci. Biobehav. Rev..

[12] Riva F. (2018). Age-related differences in the neural correlates of empathy for pleasant and unpleasant touch in a female sample. Neurobiol. Aging.

[13] Meyer M.L. (2015). Differential neural activation to friends and strangers links interdependence to empathy. Culture and Brain.

[14] Schilbach L. (2013). Toward a second-person neuroscience. Behav. Brain Sci..

[15] Redcay E., Schilbach L. (2019). Using second-person neuroscience to elucidate the mechanisms of social interaction. Nat. Rev. Neurosci..

[16] Toppi J., Siniatchkin M., Vogel P., Freitag C., Astolfi L., Ciaramidaro A. (2022). A novel approach to measure brain-to-brain spatial and temporal alignment during positive empathy. Sci. Rep..

[17] Anders S., Verrel J., Haynes J.-D., Ethofer T. (2020). Pseudo-hyperscanning shows common neural activity during face-to-face communication of affect to be associated with shared affective feelings but not with mere emotion recognition. Cortex.

[18] Xu L. (2020). Inter-subject phase synchronization differentiates neural networks underlying physical pain empathy. Soc. Cognit. Affect Neurosci..

[19] Lin J.-F.L. (2022). Dual-MEG interbrain synchronization during turn-taking verbal interactions between mothers and children. Cerebr. Cortex.

[20] Zivan M., Gashri C., Habuba N., Horowitz-Kraus T. (2022). Reduced mother-child brain-to-brain synchrony during joint storytelling interaction interrupted by a media usage. Child Neuropsychol..

[21] Ahn S. (2018). Interbrain phase synchronization during turn‐taking verbal interaction—a hyperscanning study using simultaneous EEG/MEG. Hum. Brain Mapp..

[22] Liu L. (2020). Auditory–articulatory neural alignment between listener and speaker during verbal communication. Cerebr. Cortex.

[23] Pérez A., Davis M.H. (2023). Speaking and listening to inter-brain relationships. Cortex.

[24] Watanabe H. (2022). Construction of a fiber-optically connected MEG hyperscanning system for recording brain activity during real-time communication. PLoS One.

[25] Djalovski A., Dumas G., Kinreich S., Feldman R. (2021). Human attachments shape interbrain synchrony toward efficient performance of social goals. Neuroimage.

[26] Gamliel H.N., Nevat M., Probolovski H.G., Karklinsky M., Han S., Shamay-Tsoory S.G. (2021). Inter-group conflict affects inter-brain synchrony during synchronized movements. Neuroimage.

[27] Long Y. (2022). Interpersonal conflict increases interpersonal neural synchronization in romantic couples. Cerebr. Cortex.

[28] Bavelas J.B., Coates L., Johnson T. (Dec 2000). Listeners as co-narrators. J. Pers. Soc. Psychol..

[29] Rogers C.R. (1957). The necessary and sufficient conditions of therapeutic personality change. J. Consult. Psychol..

[30] Niven K., Totterdell P., Holman D. (Aug 2009). A classification of controlled interpersonal affect regulation strategies. Emotion.

[31] Morelli S.A., Lieberman M.D., Zaki J. (2015). The emerging study of positive empathy. Social and Personality Psychology Compass.

[32] Decety J., Norman G.J., Berntson G.G., Cacioppo J.T. (Jul 2012). A neurobehavioral evolutionary perspective on the mechanisms underlying empathy. Prog. Neurobiol..

[33] Tucker D.M., Luu P., Derryberry D. (2005). Love hurts: the evolution of empathic concern through the encephalization of nociceptive capacity. Dev. Psychopathol..

[34] Perry D., Hendler T., Shamay-Tsoory S.G. (2012). Can we share the joy of others? Empathic neural responses to distress vs joy. Soc. Cognit. Affect Neurosci..

[35] Wei Y. (2023). Happy storytelling promotes emotional contagion and interpersonal closeness. Curr. Psychol..

[36] Li Y., Chen M., Zhang R., Li X. (2022). Experiencing happiness together facilitates dyadic coordination through the enhanced interpersonal neural synchronization. Soc. Cognit. Affect Neurosci..

[37] Xie E. (2021). Sharing happy stories increases interpersonal closeness: interpersonal brain synchronization as a neural indicator. Eneuro.

[38] Morelli S.A., Rameson L.T., Lieberman M.D. (Jan 2014). The neural components of empathy: predicting daily prosocial behavior. Soc. Cognit. Affect Neurosci..

[39] Lamm C., Silani G., Singer T. (Sep 2015). Distinct neural networks underlying empathy for pleasant and unpleasant touch. Cortex.

[40] Taiwo Z., Bezdek M., Mirabito G., Light S.N. (Jan 2021). Empathy for joy recruits a broader prefrontal network than empathy for sadness and is predicted by executive functioning. Neuropsychology.

[41] Stietz J., Jauk E., Krach S., Kanske P. (2019). Dissociating empathy from perspective-taking: evidence from intra-and inter-individual differences research. Front. Psychiatr..

[42] Kelly J.R., Iannone N.E., McCarty M.K. (2016). Emotional contagion of anger is automatic: an evolutionary explanation. Br. J. Soc. Psychol..

[43] N. Mackes et al., "Tracking emotions in the brain – revisiting the empathic accuracy task," Neuroimage*,* vol. 178, 06/01 2018, doi: 10.1016/j.neuroimage.2018.05.080. 10.1016/j.neuroimage.2018.05.080 PMC6057276 29890323

[44] Brett J.D., Becerra R., Maybery M.T., Preece D.A. (2023). The psychometric assessment of empathy: development and validation of the Perth Empathy Scale. Assessment.

[45] Clark M.A., Robertson M.M., Young S. (2019). “I feel your pain”: a critical review of organizational research on empathy. J. Organ. Behav..

[46] Decety J., Jackson P.L. (2004). The functional architecture of human empathy. Behav. Cognit. Neurosci. Rev..

[47] Jankowiak-Siuda K., Rymarczyk K., Grabowska A. (2011). How we empathize with others: a neurobiological perspective. Med. Sci. Mon. Int. Med. J. Exp. Clin. Res.: international medical journal of experimental and clinical research.

[48] Kogler L., Müller V.I., Werminghausen E., Eickhoff S.B., Derntl B. (2020). Do I feel or do I know? Neuroimaging meta-analyses on the multiple facets of empathy. Cortex.

[49] Tremblay M.B. (Aug 2021). I can but I shall not always Be empathic. Psychol. Rep..

[50] Rum Y., Perry A. (2020). Empathic accuracy in clinical populations. Front. Psychiatr..

[51] Kade S.A. (2024). Aberrant cognitive empathy in individuals with elevated social anxiety and regulation with emotional working memory training. Cognit. Emot..

[52] Fox N.A. (Mar 2016). Assessing human mirror activity with EEG mu rhythm: a meta-analysis. Psychol. Bull..

[53] Bekkali S., Youssef G.J., Donaldson P.H., Albein-Urios N., Hyde C., Enticott P.G. (2021). Is the putative mirror neuron system associated with empathy? A systematic review and meta-analysis. Neuropsychol. Rev..

[54] Braadbaart L., Williams J.H., Waiter G.D. (Jul 2013). Do mirror neuron areas mediate mu rhythm suppression during imitation and action observation?. Int. J. Psychophysiol..

[55] Cheng Y., Yang C.Y., Lin C.P., Lee P.L., Decety J. (May 1 2008). The perception of pain in others suppresses somatosensory oscillations: a magnetoencephalography study. Neuroimage.

[56] Arnett K., Roach A., Elzy M., Jelsone-Swain L. (Apr 2019). Childhood emotional invalidation and right hemispheric mu suppression during a pain empathy task: an EEG study. Soc. Neurosci..

[57] DiGirolamo M.A., Simon J.C., Hubley K.M., Kopulsky A., Gutsell J.N. (Oct 2019). Clarifying the relationship between trait empathy and action-based resonance indexed by EEG mu-rhythm suppression. Neuropsychologia.

[58] Gutsell J.N., Simon J.C., Jiang Y. (Oct 2020). Perspective taking reduces group biases in sensorimotor resonance. Cortex.

[59] Hobson H.M., Bishop D.V. (2017). The interpretation of mu suppression as an index of mirror neuron activity: past, present and future. R. Soc. Open Sci..

[60] Hobson H.M., Bishop D.V. (2016). Mu suppression–a good measure of the human mirror neuron system?. Cortex.

[61] Jospe K., Genzer S., Klein Selle N., Ong D., Zaki J., Perry A. (Nov 2020). The contribution of linguistic and visual cues to physiological synchrony and empathic accuracy. Cortex.

[62] Ansfield M.E. (2007). Smiling when distressed: when a smile is a frown turned upside down. Pers. Soc. Psychol. Bull..

[63] Schreiter S., Pijnenborg G.H., Aan Het Rot M. (Aug 15 2013). Empathy in adults with clinical or subclinical depressive symptoms. J. Affect. Disord..

[64] Habermas T., Ott L.M., Schubert M., Schneider B., Pate A. (2008). Stuck in the past: negative bias, explanatory style, temporal order, and evaluative perspectives in life narratives of clinically depressed individuals. Depress. Anxiety.

[65] Baron-Cohen S., Wheelwright S. (2004). The empathy quotient: an investigation of adults with Asperger syndrome or high functioning autism, and normal sex differences. J. Autism Dev. Disord..

[66] Beck A.T. (1979).

[67] Chen Y.R. (2009). Master Thesis, Department of Education.

[68] Ma W.-Y., Shih Y.-Y. (24 August 2018). *Proceedings of the Eleventh International Conference on Language Resources and Evaluation (LREC 2018)*, Miyazaki, Japan.

[69] Rottenberg J., Ray R., Gross J., Coan J., Allen J. (2007). The Handbook of Emotion Elicitation and Assessment.

[70] Miranda R., Kihlstrom J. (2005). Mood congruence in childhood and recent autobiographical memory. Cognit. Emot..

[71] Eerola T., Vuoskoski J.K. (2011). A comparison of the discrete and dimensional models of emotion in music. Psychol. Music.

[72] Bradley M.M., Cuthbert B.N., Lang P.J. (1996). Picture media and emotion: effects of a sustained affective context. Psychophysiology.

[73] Delorme A., Makeig S. (2004). EEGLAB: an open source toolbox for analysis of single-trial EEG dynamics including independent component analysis. J. Neurosci. Methods.

[74] Dong L. (2017). MATLAB toolboxes for reference electrode standardization technique (REST) of scalp EEG. Front. Neurosci..

[75] Kothe C.A.E., Jung T.-P. (2016). Artifact removal techniques with signal reconstruction. U.S. Patent Appl..

[76] Delorme A., Sejnowski T., Makeig S. (2007). Enhanced detection of artifacts in EEG data using higher-order statistics and independent component analysis. Neuroimage.

[77] Pion-Tonachini L., Kreutz-Delgado K., Makeig S. (2019). ICLabel: an automated electroencephalographic independent component classifier, dataset, and website. Neuroimage.

[78] Oberman L.M., Hubbard E.M., McCleery J.P., Altschuler E.L., Ramachandran V.S., Pineda J.A. (2005). EEG evidence for mirror neuron dysfunction in autism spectrum disorders. Cognit. Brain Res..

[79] Peled-Avron L., Goldstein P., Yellinek S., Weissman-Fogel I., Shamay-Tsoory S.G. (Jul 31 2018). Empathy during consoling touch is modulated by mu-rhythm: an EEG study. Neuropsychologia.

[80] Pascual-Marqui R.D. (2002). Standardized low-resolution brain electromagnetic tomography (sLORETA): technical details. Methods Find Exp. Clin. Pharmacol..

[81] Tadel F., Baillet S., Mosher J.C., Pantazis D., Leahy R.M. (2011). Brainstorm: a user-friendly application for MEG/EEG analysis. Comput. Intell. Neurosci..

[82] Joshi A.A. (2022). A hybrid high-resolution anatomical MRI atlas with sub-parcellation of cortical gyri using resting fMRI. J. Neurosci. Methods.

[83] Zaki J., Bolger N., Ochsner K. (Aug 2009). Unpacking the informational bases of empathic accuracy. Emotion.

[84] Simon J.C., Gutsell J.N. (May 4 2021). Recognizing humanity: dehumanization predicts neural mirroring and empathic accuracy in face-to-face interactions. Soc. Cognit. Affect Neurosci..

[85] Kraus M.W. (Oct 2017). Voice-only communication enhances empathic accuracy. Am. Psychol..

[86] Gu X., Han S. (May 15 2007). Attention and reality constraints on the neural processes of empathy for pain. Neuroimage.

[87] Henry J.D., Grainger S.A., von Hippel W. (Jan 18 2023). Determinants of social cognitive aging: predicting resilience and risk. Annu. Rev. Psychol..

[88] Rameson L.T., Morelli S.A., Lieberman M.D. (2012). The neural correlates of empathy: experience, automaticity, and prosocial behavior. J. Cognit. Neurosci..

[89] Hiraoka D., Nomura M. (Jun 16 2016). The influence of cognitive load on empathy and intention in response to infant crying. Sci. Rep..

[90] Bajouk O., Hansenne M. (Dec 2019). Dispositional perspective-taking and empathic concern modulate the impact of cognitive load on empathy for facial emotions. Psychol. Rep..

[91] Carr L., Iacoboni M., Dubeau M.-C., Mazziotta J.C., Lenzi G.L. (2003). Neural mechanisms of empathy in humans: a relay from neural systems for imitation to limbic areas. Proc. Natl. Acad. Sci. USA.

[92] Elliot A.J. (2006). The hierarchical model of approach-avoidance motivation. Motiv. Emot..

[93] Lane R.C., Koetting M.G., Bishop J. (2002). Silence as communication in psychodynamic psychotherapy. Clin. Psychol. Rev..

[94] Kacperck L. (1997). Non-verbal communication: the importance of listening. Br. J. Nurs..

[95] Capretto P. (Feb 2015). Empathy and silence in pastoral care for traumatic grief and loss. J. Relig. Health.

[96] Bombari D., Schmid Mast M., Brosch T., Sander D. (2013). How interpersonal power affects empathic accuracy: differential roles of mentalizing vs. mirroring?. Front. Hum. Neurosci..

[97] Zaki J. (May 2013). Cue integration: a common framework for social cognition and physical perception. Perspect. Psychol. Sci..

[98] Arioli M., Crespi C., Canessa N. (2018). Social cognition through the lens of cognitive and clinical neuroscience. BioMed Res. Int..

[99] Shamay-Tsoory S. (2015). The neuropsychology of empathy: evidence from lesion studies. Rev. Neuropsychol..

[100] Bellou P., Gerogianni K. (2007). The contribution of family in the care of patient in the hospital. Health Sci. J..

[101] Collins H.K., Minson J.A., Kristal A., Brooks A.W. (2024). Conveying and detecting listening during live conversation. J. Exp. Psychol. Gen..

[102] Oertel C., Jonell P., Kontogiorgos D., Mora K.F., Odobez J.-M., Gustafson J. (2021). Towards an engagement-aware attentive artificial listener for multi-party interactions. Frontiers in Robotics and AI.

[103] Cook J.M., Biyanova T., Elhai J., Schnurr P.P., Coyne J.C. (2010). What do psychotherapists really do in practice? An Internet study of over 2,000 practitioners. Psychother. Theor. Res. Pract. Train..

[104] Pletzer J.L., Sanchez X., Scheibe S. (2015). Practicing psychotherapists are more skilled at downregulating negative emotions than other professionals. Psychotherapy.

