# Supplementary material for: Feedback matters: EEG correlates of empathy involved in the naturalistic communication of emotions
Source: Heliyon. 2024 Sep 27;10(19):e38574. doi: 10.1016/j.heliyon.2024.e38574 (PMC11472091; doi:10.1016/j.heliyon.2024.e38574)
Supplement: Multimedia component 1 [file mmc1.pdf]

## Supplementary

***Empathic accuracy, emotional contagion, and emotional intensity***

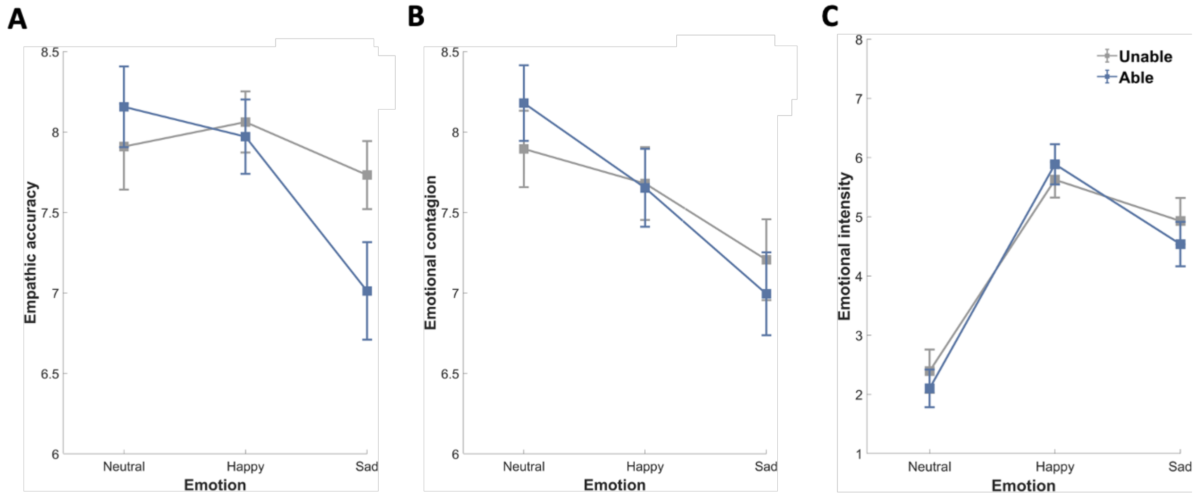


Supplementary Fig. S1. Behavioral results: (A) Empathic accuracy, where higher values indicate the accuracy with which the empathizer characterized the emotional state of the target; (B) Emotional contagion, where higher values indicate the closeness with which the empathizer resonated with the emotional state of the target; (C) Emotional intensity, where values indicate the level of intensity of the empathizer experienced. Data points indicate the mean (±SE) value for a given (color-coded) feedback condition.

Supplementary Table S1. Results (main effect and interaction) of two-way repeated measures ANOVA using feedback (unable, able) and emotion (neutral, happy, sad) as within-subjects factors.

| | **Two-way ANOVA** | | | | | |
|---|---|---|---|---|---|---|
| | **Feedback** | | **Emotion** | | **Feedback×Emotion** | |
| | F-value | P-value | F-value | P-value | F-value | P-value |
| **EA** | 3.083 | 0.088 | 5.577 | **0.006**$^{**}$ | 4.412 | **0.016**$^{*}$ |
| **EC** | 0.014 | 0.906 | 7.226 | **0.001**$^{**}$ | 1.266 | 0.289 |
| **EI** | 0.856 | 0.362 | 71.139 | **0.001**$^{**}$ | 2.047 | 0.137 |

Note: EA, empathic accuracy; EC, emotional contagion; EI, emotional intensity. $^{*}$P < 0.05; $^{**}$P < 0.01.

Supplementary Table S2. Results (post-hoc) of two-way repeated measures ANOVA using feedback (unable, able) and emotion (neutral, happy, sad) as within-subjects factors.

| | Post-hoc | | | | | | | |
|---|---|---|---|---|---|---|---|---|
| | **Neutral-Happy** | | **Neutral-Sad** | | **Happy-Sad** | | **Unable-Able** | |
| | Diff | P-value | Diff | P-value | Diff | P-value | Diff | P-value |
| **EA** | 0.016 | 1.000 | 0.660 | 0.052 | 0.644 | **0.001**$^{**}$ | 0.188 | 0.088 |
| **EC** | 0.371 | 0.617 | 0.937 | **0.006**$^{**}$ | 0.566 | **0.003**$^{**}$ | -0.016 | 0.906 |
| **EI** | -3.509 | **0.001**$^{**}$ | -2.487 | **0.001**$^{**}$ | 1.022 | **0.001**$^{**}$ | 0.139 | 0.362 |

Note: EA, empathic accuracy; EC, emotional contagion; EI, emotional intensity; Diff, difference. $^{**}P < 0.01$.
